# The effect of subjective sleep latency on BMI of medical interns during and before COVID-19 pandemic

**DOI:** 10.5935/1984-0063.20200112

**Published:** 2021

**Authors:** Ehsan Bastanhagh, Reza Erfanian

**Affiliations:** 1 Department of Anesthesiology and Critical Care, Tehran University of Medical Sciences, Tehran, Iran.; 2 Department of Otorhinolaryngology-Head and Neck Surgery, Sleep Medicine Fellowship, Otorhinolaryngology Research Center, Amir Alam Hospital, Tehran University of Medical Sciences, Tehran, Iran.

**Keywords:** Coronavirus Infections, Sleep Latency, Obesity

## Abstract

**Objectives:**

Longer subjective sleep latency and eveningness chronotype are associated with higher BMI. Moreover, COVID-19 lockdown changes have been associated with increased BMI. The aim of this study was to investigate the effect of subjective sleep parameters on BMI of medical interns during and before COVID-19 pandemic.

**Material and Methods:**

This cross-sectional study was performed among medical interns. Bedtime, sleep latency, waking time, sleep duration, and reduced morningness-eveningness scores were evaluated.

**Results:**

There was significant difference between bedtime before (00:11±50) and during (01:10±85) the pandemic in females (p<0.001). The mean circadian score before and during the pandemic showed significant decrease in females (p=0.011). The correlation between BMI with subjective sleep latency in females before and during the pandemic ((r=0.439, p=0.017), (r=0.422, p=0.014)) was significant.

**Conclusion:**

COVID-19 pandemic was associated with a change toward nocturnal life among female medical interns. Subjective sleep latency was significantly correlated with BMI in females.

## INTRODUCTION

Recently, the prevalence of obesity has been increased swiftly in both developed and developing countries. It seems that the continuation of the rapid rise in obesity rate would culminate in an estimated global prevalence of 18% in men and more than 21% in women by 2025. However, considering the social stratification, the change in prevalence of obesity is not merely connected with the level of income or wealth^1^.

Multiple risk factors and complex mechanisms further the development and persistence of obesity. An effective factor in regulating metabolism is circadian rhythm as it regulates energy metabolism and enhances the strength of certain dynamic activities during day and night. Aside from common risk factors, two determining influences for obesity and other metabolic disorders are sleep and circadian disruptions, and their impact can be eliminated by modifying them^2^.

For preserving homeostasis, the body constantly adapts to both psychological and physiological stressors. However, constant exposure to stressful situations is often associated with metabolic imbalance. Acute or unexpected stressors reduce body weight and food intake, while chronic social or predictable stressors are associated with increased caloric intake and weight gain^3^. Moreover, chronic stress has negative impacts on vital organs of the body including the physiological processes, namely fat distribution, adiposity, and eating behavior while the acute stress can be alleviated^4^.

On December 31, 2019, many cases of infection with COVID-19 were reported in Wuhan, China. Due to unabated transmission of COVID-19, it spread rapidly throughout the world and became pandemic in a matter of few days. To help limit the spread of COVID-19, quarantine and segregation were among the proposed options, which led to a change in lifestyle, thereby affecting the entire living conditions, including the mental health of the community^5^. In this regard, the findings of the study conducted by Zhang et al. (2020)^6^ showed that the psychological stress caused by COVID-19 has an impact on the overall health, sleep quality, and stress of people in the community. In fact, mental disorders are usually accompanied by sleep disturbances and changes in the sleep cycle and circadian rhythm^7^.

In the study by Cellini et al. (2020)^8^, the results showed that during the COVID-19 pandemic, the use of digital media before bedtime ramped up, sleep quality decreased, and the number of people with higher levels of stress and depression increased. Also, the results of another study showed that nighttime vigilance is associated with a decrease in sleep quality in medical students, and it is interesting to note that during COVID-19 pandemic, the rate of nocturnal chronotypes increased^9^.

Studies have also shown that one of the causes of obesity is circadian rhythm sleep disorders because many physiological functions of the human body have periodic changes that are repeated almost every 24 hours. The circadian rhythm is regulated very slowly in the human body, so irregular working hours as well as changes in sleep patterns and daily activities result in improper coordination of internal physiological processes and rhythms with external stimuli, thus leading to physical and mental problems^10^. Moreover, the results of epidemiological studies indicate an association between inadequate sleep and an increased risk of obesity^11^.

The gender has important effect on correlations between BMI and sleep parameters. The results of St-Onge et al. (2010)^12^ study showed BMI was inversely associated with sleep duration in women and in men in which associations though not statistically significant were in the same direction and of similar magnitude. In a sample collected from Southern France, sleep duration had no association with BMI in men but significant relationship was found in women^13^.

One study indicated that after admission to college, students reduced their sleep in order to manage their heavy workloads and among them, medical students were extremely accustomed to unorganized and irregular sleeping patterns. The findings proved the high rate of poor subjective sleep quality in all students studying at different years in undergraduate medical course^14^. Ayala et al. (2017)^15^ show that irregular sleep patterns may contribute to fatigue in medical students even when duration of sleep seems to be adequate.

As the obesity is becoming a major worldwide problem, and is associated with sleep disturbance, we would examine the relationship between BMI and subjective sleep parameters in young medical interns. In addition, we examined consistency of this association against stress and workload change which imposed by COVID-19 pandemics in this peculiar population.

## MATERIAL AND METHODS

This cross-sectional study was performed among medical interns who were graduation candidates. The study was conducted at two time intervals of August 2019 and August 2020 on medical interns. Using convenient sampling, 79 medical interns were selected in August 2019 and 73 in August 2020. The study tools included age, sex, body mass index (BMI), bedtime, subjective sleep latency, sleep time, wake time, circadian score, and circadian type. Chronotype was also assessed by the reduced morningness-eveningness-questionnaire (rMEQ)^16^. The rMEQ contains 5 multiple choice questions each having four or five response options about individual’s rising and bedtimes, preferred time for physical and mental performance, and alertness after rising and before going to bed. The participants were categorized with scores from high to low into the following sub-types: morning types (M-types), intermediate or neither types (N-types), and evening types (E-types). The Persian version of the questionnaire showed acceptable reliability and concurrent validity^17^. The study’s protocol was approved by the local ethical research committee and written informed consent was obtained from the medical school principal and all participants. Statistical analysis was done using SPSS software (Version 23.0, IBM Corporation, Armonk, N.Y., USA). The normal distribution of the data was checked by the Kolmogorov-Smirnov test and accordingly data were analyzed through performing Mann-Whitney U test and Spearman’s correlation. All procedures and data comparisons were carried out in compliance with relevant laws and guidelines and in accordance with the ethical standards of the declaration of Helsinki.

## RESULTS

The mean ages of male participants before COVID-19 and during it were 26.26±0.98 and 26.18±1.04 years, respectively (*p*=0.965) and 26.11±1.9 and 26.22±0.91 years in females (*p*=0.306). Before and during COVID-19 pandemic, 54.4% and 53.4% of participants were females, respectively (*p*=0.901). BMI in males before and during the pandemic were 23.46±3.14 and 25.05±3.43, respectively (*p*=0.091) and in females were 23.10±3.43 and 23.18±4.23, respectively (*p*=0.768).

The frequency of each circadian type before COVID-19 was 25 (31.6%) for E-type, 45 (57.0%) for N-type, and 9 (11.4%) for M-type and after pandemic, 34 (46.6%) for E-type, 34 (46.6%) for N-type, and 5 (6.8%) for M-type (*p*=0.148).

The mean of circadian rhythm of medical interns before and during the COVID-19 pandemic showed a significant decrease among females (*p*=0.011) but this score was not statistically significant in males before and during the pandemic (*p*=0.494) (Table 1).

**Table 1:** Comparison of sleep status in male and female medical students before and after COVID-19 pandemic.

Variable		Mean±SD		*p*-value
		Before	After	
Bedtime (hr)	Male	00:54±74’	01:10±85’	0.540
	Female	00:11±50’	01:15±85’	<0.001
Sleep latency (min)	Male	19.19±15.53	25.45±23.38	0.326
	Female	21.53±17.61	35.40±30.70	0.061
Wake time	Male	06:54±82’	07:57±120’	0.024
	Female	06:48±60’	08:15±96’	<0.001
Sleep time (min)	Male	357.39±92.20	358.59±117.54	0.400
	Female	349.68±82.28	362.08±120.56	0.501
Circadian score	Male	12.72±3.98	12.03±3.94	0.494
	Female	13.98±3.21	11.96±3.02	0.011

The results of the present study showed that the difference between the mean of medical interns’ bedtime before and during the COVID-19 pandemic was not statistically significant in males (*p*=0.540) but the bedtime mean during the COVID-19 pandemic increased significantly in females (*p*<0.001). Although subjective sleep latency and sleep time mean of medical interns increased in both males and females during the COVID-19 pandemic, this difference was not statistically significant (*p*>0.05). Wake time means of medical interns in both males and females showed a significant increase during the pandemic, and this increase was more among females rather than males (*p*=0.024 and *p*<0.001, respectively) (Table 1).

The correlation between body mass index (BMI) with sleep parameters and circadian rhythm before and during the pandemic showed that there was a positive and significant correlation between subjective sleep latency and body mass index (BMI) in females before the pandemic (r=0.439, *p*=0.017) and similarly during the pandemic, this positive significant relationship was still observed (r=0.422, *p*=0.014). There was no significant correlation between circadian rhythm and other sleep parameters with body mass index before and during the pandemic in females (Table 2).

**Table 2:** The correlation of sleep parameters and BMI in male and female medical interns before and during COVID-19 pandemic.

Variable		Before (Pearson correlation, p- value)	During (Pearson correlation, p- value)
Bedtime	Male	r=0.220, p=0.261	r=0.287, p=0.117
	Female	r=0.134, p=0.481	r=-0.059, p=0.744
Subjective sleep latency	Male	r=-0.373, p=0.051	r=0.165, p=0.367
	Female	r=0.439, p=0.017	r=0.422, p=0.014
Wake time	Male	r=0.192, p=0.328	r=0.142, p=0.439
	Female	r=0.104, p=0.592	r=0.327, p=0.068
Sleep time	Male	r=0.016, p=0.943	r=0.206, p=0.259
	Female	r=-0.272, p=0.153	r=-0.055, p=0.774
Circadian score	Male	r=-0.348, p=0.069	r=-0.117, p=0.525
	Female	r=-0.241, p=0.192	r=0.061, p=0.730

## DISCUSSION

The results of the present study show that the circadian rhythm score during the COVID-19 pandemic compared to the same period last year among female medical interns had a significant increase towards nocturnal life and bedtimes and wake times were later than before. Interns were lying in the bed longer to fall asleep, possibly due to the adverse effects of the COVID-19 pandemic.

However, the sleep time did not change, which could be due to the partial closure of hospital wards and having more free time to sleep. Epidemiological studies have demonstrated a higher prevalence of insomnia symptoms in females in comparison to males. The study by Song et al. (2020)^18^ showed that during the COVID-19 pandemic, women suffered more stress than men, and also, the stressful condition was more frequent among female individuals under 45, which is in line with the findings of the present study. It has been shown that the chronotype is a relatively stable and currently considered as a trait-like characteristic^19^. However, chronotype can be changed by environmental zeitgebers (e.g., seasonal chronotype change)^20^. In our study, we found no change in the chronotype of men interns but delayed phase shift in women interns. It can be explained as young women are more morning-oriented than men^21^ and any change in environmental zeitgebers in favor of evening has a larger effect on women according to “phase response curve for light” rule^22^.

The women chronotype change toward evening is more important than men regarding to depression risk, which make the COVID-19 pandemics psychological impact more complicated^23^. We observed a borderline positive association between subjective sleep latency and BMI in men, which may be due to nighttime exercises as it could be explained both reduced BMI and longer sleep latency as these special populations would have time for exercise just in the evening before pandemics.

Moreover, the current study revealed a significant correlation between subjective sleep latency and BMI in females; likewise, Bowman et al. (2019)^24^ demonstrated the significant relationship between subjective sleep latency, BMI, and metabolic syndrome. This may be due to a complex relationship between sleep patterns and human metabolism, and sleep may play a pivotal role in weight homeostasis.

Students, particularly medical interns, are vulnerable to sleep disorders such as insomnia, constant night wakings, falling asleep late, and poor quality sleep, and these disorders have a major effect on their quality of life and education. The eating behavior change caused by environmental stress^25^ and the association of BMI and sleep parameters^12^ is different between men and women, besides sleep changes by stress is more robust in women^26^. Our data supported that sleep parameters changes just occurred in women and the association between subjective sleep latency and BMI was only observed in women.

One of the limitations of this study was the small sample size and access to only medical interns, which limited the evaluation of students from different healthcare disciplines. Although we calculated BMI by self-reported weight and height it has been shown that self-reported weight and height have good agreement with actual measurements in young adults^27^. The fluctuation in weight is well-known phenomena^28^, which may bias measured data as well as self-reported data.

## CONCLUSION

COVID-19 pandemic was associated with a change toward nocturnal life among female medical interns. Subjective sleep latency was significantly correlated with BMI in females and COVID-19 pandemics and its related life changes did not affect this association. For a better interpretation of this association, prospective and interventional studies are needed.
